# Decoding Mast Cell-Microglia Communication in Neurodegenerative Diseases

**DOI:** 10.3390/ijms22031093

**Published:** 2021-01-22

**Authors:** Jagdeep K. Sandhu, Marianna Kulka

**Affiliations:** 1 Human Health Therapeutics Research Centre, National Research Council Canada, 1200 Montreal Road, Ottawa, ON K1A 0R6, Canada; 2 Department of Biochemistry, Microbiology and Immunology, University of Ottawa, 451 Smyth Road, Ottawa, ON K1H 8M5, Canada; 3 Nanotechnology Research Centre, National Research Council Canada, 11421 Saskatchewan Drive, Edmonton, AB T6G 2M9, Canada; 4 Department of Medical Microbiology and Immunology, University of Alberta, Edmonton, AB T6G 2E1, Canada

**Keywords:** innate immune system, mast cell mediators, degranulation, cytokines, chemokines, microglia, therapeutics, communication, NLRP3 inflammasome, neuroinflammation

## Abstract

Microglia, resident immune cells of the central nervous system (CNS), play a pivotal role in immune surveillance and maintenance of neuronal health. Mast cells are also important resident immune cells of the CNS but they are underappreciated and understudied. Both microglia and mast cells are endowed with an array of signaling receptors that recognize microbes and cellular damage. As cellular sensors and effectors in the CNS, they respond to many CNS perturbations and have been implicated in neuroinflammation and neurodegeneration. Mast cells contain numerous secretory granules packaged with a plethora of readily available and newly synthesized compounds known as ‘mast cell mediators’. Mast cells act as ‘first responders’ to a pathogenic stimuli and respond by degranulation and releasing these mediators into the extracellular milieu. They alert other glial cells, including microglia to initiate neuroinflammatory processes that culminate in the resolution of injury. However, failure to resolve the pathogenic process can lead to persistent activation, release of pro-inflammatory mediators and amplification of neuroinflammatory responses, in turn, resulting in neuronal dysfunction and demise. This review discusses the current understanding of the molecular conversation between mast cells and microglia in orchestrating immune responses during two of the most prevalent neurodegenerative diseases, namely Alzheimer’s disease and Parkinson’s disease. Here we also survey the potential emerging therapeutic approaches targeting common pathways in mast cells and microglia to extinguish the fire of inflammation.

## 1. Introduction

The most common pathological manifestations of age-related neurodegenerative diseases are the extracellular accumulation of misfolded aggregated proteins, such as β-amyloid and α-synuclein, and a progressive loss of vulnerable populations of neurons in specific brain regions [1]. Alzheimer’s disease (AD) is the most common neurodegenerative disease that is characterized by the presence of plaques composed of β-amyloid protein and neurofibrillary tangles composed of hyper-phosphorylated tau protein [2]. Parkinson’s disease (PD) is the world’s second most prevalent neurodegenerative disease and is associated with a loss of dopaminergic neurons in the substantia nigra and accumulation of aggregated α-synuclein [3]. Other common hallmarks of these diseases are abnormalities in the ubiquitin-proteasomal and autophagosomal or lysosomal system, mitochondrial dysfunction, and oxidative stress. Histopathological evidence of activated microglia surrounding β-amyloid and α-synuclein deposition in the brain tissues from AD and PD patients has been well documented [4,5]. It has become increasingly evident that inflammation of the nervous system, termed ‘neuroinflammation’, is the main culprit of most, if not all, neurodegenerative diseases. Neuroinflammation occurs in the absence of an infectious agent and may be triggered by the accumulation of proteins with abnormal conformations (e.g., β-amyloid and α-synuclein), signals produced by injured neurons, or imbalances between pro- and anti-inflammatory processes [6,7]. The precise relationship between neuroinflammation and the pathogenesis of neurodegenerative diseases remains unclear and is the subject of ongoing research. In recent years much attention has been paid to understand the role of glial cells, namely microglia and astroglia, in the maintenance of neuronal survival and the pathogenesis of several neurodegenerative diseases, including AD and PD [8].

Microglia are the archetypal innate immune cells of the CNS and have been extensively studied for their role in protecting the brain against infectious and non-infectious sterile tissue injury [9]. Activated microglial cells are an important component of neuroinflammation where they are involved not only in the maintenance of normal brain health but also play a central role in the initiation and progression of neurodegenerative diseases [10,11]. More recently, a greater focus has been placed on mast cells as modulators of neuroinflammation and neurodegeneration [12,13,14]. Mast cells are armed with an arsenal of preformed and preactivated pro-inflammatory mediators that can be immediately deployed for battle against infection and tissue damage [15]. There is an increasing appreciation for the active communication between mast cells and microglia and these two cell types of the innate immune system have been described as the two tracks on the road to neuroinflammation and neurodegeneration [16]. In the sections below, we discuss the molecular mechanisms underlying the crosstalk between mast cells and microglia and their impact on neuroinflammation and neurodegeneration.

## 2. Innate Immune System of the Brain

The innate immune system is the first line of defense that protects the CNS against exogenous pathogens, including viruses, bacteria, fungi, protozoans and parasites and endogenous sterile molecules, including abnormal misfolded forms of proteins such as β-amyloid and α-synuclein [17]. Both microglia and mast cells are equipped with a repertoire of innate immune sensors that enable them to recognize conserved pathogen motifs via pattern-recognition receptors (PRRs). The innate immune system is comprised of a sensing apparatus that has evolved to detect “stranger” signals from microbes and “danger” signals from damaged cells. The PRRs comprise a large family of receptors, including toll-like receptors (TLR), nucleotide-binding and oligomerization domain (NOD)-like receptors (NLRs), and retinoic acid-inducible gene-I-like receptors (RIG-I-like receptors, RLRs). TLRs are mainly present on plasma membrane and endocytic vesicles whereas NLRs and RLRs are present in the cytosol [18,19]. Other receptors, such as scavenger receptors (SRs), C-type lectin receptors (CLRs), and complement receptors (CRs), are also involved in sensing microbes and tissue damage. A detailed description of these pathways can be found in recent reviews [11,20,21].

The PRRs—cooperate to survey the extracellular and intracellular environment and act as chemosensors of pathogens and sterile damage [22]. Ligation of TLRs initiates an intracellular signaling cascade that culminates in the secretion of inflammatory mediators, such as interleukins (IL-1β, IL-6), interferon-γ and tumor necrosis factor-alpha (TNF-α), chemokines ((macrophage inflammatory protein-1 (MIP-1), monocyte chemoattractant protein-1 (MCP-1)), acute-phase proteins, cell adhesion molecules and oxidative stress-related enzymes thereby triggering protective immune responses and tissue repair [18,23]. Exogenous microbial pathogen-associated molecular patterns (PAMP) or endogenous danger-associated molecular patterns (DAMP) activate the innate immune cells by engaging TLRs. For example, the bacterial PAMP, lipopolysaccharide (LPS), binds TLR4/CD14 complexes and activates signaling pathways to initiate localized inflammatory responses. Sterile damage in the CNS in the form of neurodegeneration-associated molecular patterns (NAMP), similar to PAMPs and DAMPs in the peripheral immune system, similarly activates neuroinflammation and neurodegeneration. The exact identity of the NAMPs that initiate this process is poorly understood but likely includes fibrillar β-amyloid, α-synuclein, or constituents of dying neurons, such as extracellular purines (adenosine triphosphate, ATP) [24], mitochondrial DNA, high-mobility group box protein 1 (HMGB1), lysophosphatidylcholine [25], S100 proteins and heat shock proteins (HSPs) [19]. Since NAMPs (e.g., β-amyloid and α-synuclein) contribute to neuroinflammation and may initiate the progression of neurodegenerative diseases such as AD and PD, the underlying cellular mechanisms involved in these processes is receiving increased attention.

## 3. Cellular Sentinels of the Brain

### 3.1. Mast Cells

**Figure 1:**
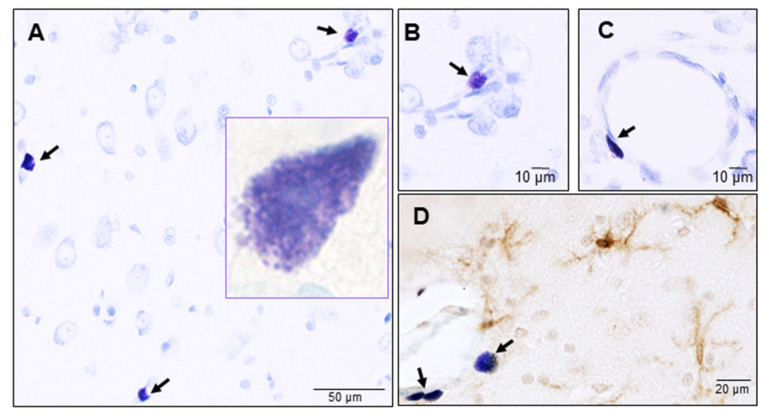
Mast cells in the naïve rat brain. Mast cells were identified by toluidine blue staining and their typical metachromasia. (**A**) Mast cells at the level of thalamus, inset shows a high magnification view of a granulated parenchymal mast cell; (**B**) A roundish mast cell around a small blood capillary; (**C**) An elongated mast cell in close proximity to a larger blood capillary; (**D**) Mast cells in close association with microglia (identified by an antibody against the ionized calcium binding adaptor molecule-1 (Iba-1) brown stain) in the brain parenchyma. Arrows indicate toluidine blue stained mast cells. Shown in panel D is a roundish mast cell and two elongated mast cells in close proximity to a blood capillary.

Mast cells were first described by Paul Ehrlich, a noble prize winner in 1876 who named them “Mastzelle”, which in German means “well-fed” cells [26]. Mast cells are derived from myeloid hematopoietic stem cells in the bone marrow. They leave the bone marrow as early CD34+ progenitors, then circulate in the blood and fully mature only when they migrate to the tissues where they undergo terminal differentiation and maturation [27]. If all of the mast cells in the body were collected into a single organ, it would likely be the size of a spleen. Unlike human skin, lung, and gut mast cells, we know very little about brain mast cells [28]. It appears that, compared to the skin and gut, there are relatively few mast cells in the brain [29]. Yet, despite their small numbers, brain mast cells appear to be dynamic cells that are continuously redistributing throughout the brain and reacting to internal and external stimuli [29]. Mast cells are typically found around the third ventricle, choroid plexus and the leptomeninges. In the CNS parenchyma, they reside mainly in the hippocampus and thalamic hypothalamic region (Figure 1A) [29,30,31]. They have also been observed posterma in the medulla [32]. The region specific localization of brain mast cells suggests that their migration might be regulated by cytokines, chemokines, neuropeptides and growth factors, such as nerve growth factor (NGF).

#### Mast Cells: Guardians of the Gateway to the Brain

Mast cells are strategically positioned to act as sentries in the brain. They are typically situated close to the neurovascular unit on the brain side of the blood-brain barrier (BBB) (Figure 1B,C) and are found in close proximity to microglial cells (Figure 1D) [33]. Increased disruption and breakdown of the BBB allows mast cells to migrate from the blood to the brain [34]. In an elegant study, Silverman et al. showed that mature mast cells, similar to other immune cells, can cross the BBB, penetrate the CNS parenchyma and situate themselves adjacent to astrocyte end feet [34]. Human mast cells are often categorized into at least two distinct phenotypes based on their expression of the proteases (chymase and tryptase) and surface receptors such as mas-related G protein-coupled receptor (Mrgpr). However, it appears that brain mast cells constitute a third phenotype, distinguished by their expression of the chymotrypsin cathepsin G [35] and the stem cell factor receptor (c-kit). Even within the brain, mast cells display pleiotropic phenotypes. Although intracerebral mast cells lack the expression of the tyrosine kinase receptor Kit, leptomeninges are comprised of a mixed population of c-kit-positive and negative cells [29,36]. Other mediators, including neurotrophin-3 (NT-3), nerve growth factor (NGF), transforming growth factor-β (TGF-β), IL-4, and IL-9, which are produced by peripheral mast cells, could also be important for the growth and survival of brain mast cells [37]. Under normal physiological conditions, brain parenchymal mast cells lack the expression of the high affinity immunoglobulin E (IgE) receptor, also known as Fc epsilon receptor 1 (FcεR1) [38], suggesting that the CNS lacks the ability to mount IgE-dependent allergic immune responses.

The most prominent feature of all tissue mast cells is about 50–200 large lysosome-like secretory granules which are distributed throughout the cytosol. Mast cells store a sophisticated array of preformed and pre-activated inflammatory products, like vasoactive amines (e.g., histamine and serotonin), proteases, proteoglycans, cytokines and growth factors in their cytoplasmic granules [37]. Notably, as the first responders, mast cells’ secretory granules store preformed TNF-α, as well as a variety of proteases [39]. Mast cells can be activated by a wide range of immunological and non-immunological stimuli (DAMPs, such as amyloid-β and α-synuclein) and act as the cellular sensors of the innate immune system [40,41]. Although some mast cell degranulation is observed even under resting conditions, upon stimulation, mast cells can rapidly release their granule contents by either piecemeal or compound and anaphylactic degranulation [42]. During degranulation, mast cells rapidly release pro-inflammatory mediators (e.g., histamine and tryptase) within a few seconds to minutes into the extracellular milieu followed by a delayed secretion within minutes to hours to even days of de novo synthesized effector molecules, such as arachidonic acid metabolites (e.g., prostaglandins and leukotrienes), cytokines (e.g., TNF-α and IL-1β), and chemokines (e.g., chemokine (C-X-C motif) ligand 8 (CXCL8) and chemokine (C-X-C motif) ligand 10 (CXCL10)), resulting in the amplification of inflammation. Autocrine production of some of these mediators can subsequently inhibit further mast cell activation, thereby leading to resolution of the inflammatory response [43], but these mediators can also act in a paracrine manner, reaching a distance of over 50 µm from the cell body in the mouse brain. In addition to reaching distant cells and tissues via interstitial fluid and blood flow, mast cells release volatile molecules and gases that can permeate and extend even further. Mast cells produce reactive nitrogen and oxygen species (RNOS) and nitric oxide during an inflammatory response [43]. It is for these reasons, that mast cells are considered to be the first responders to pathogenic events by alerting other immune effector cells to initiate innate and adaptive immune responses. It is therefore not unreasonable to hypothesize that mast cells may play a similar role in the brain. Similar to their role in other tissues, mast cells may activate and orchestrate the behaviors of the other brain immune cells such as astroglia and microglia and thereby regulate neuroinflammatory responses in the CNS.

### 3.2. Microglia

Microglia, resident parenchymal immune cells of the CNS, are considered to be highly specialized phagocytes and are the only myeloid cells found in the healthy brain. However, unlike peripheral macrophages which are derived from the hematopoietic progenitor cells, adult microglia originate from primitive myeloid progenitors in the yolk sac and enter the developing CNS during embryogenesis [44]. As the resident professional phagocytes of the brain, microglia play a crucial role in the innate immune response of the CNS to infection or injury and are critical in the maintenance of CNS homeostasis, neuronal development, neurogenesis, and overall brain health [45]. Over the past couple of decades they have garnered much attention for their role in neuroinflammation and neurodegeneration [8].

**Figure 2:**
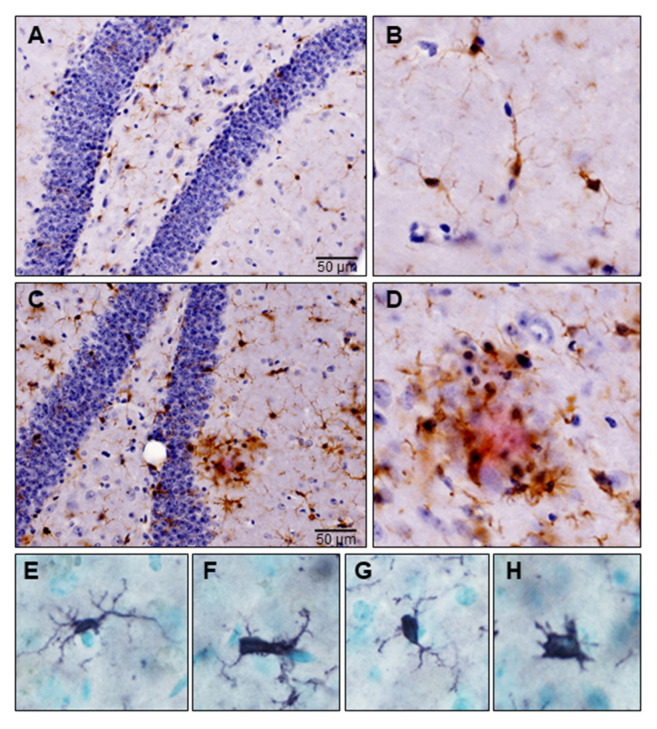
Microglia in a triple transgenic mouse model of Alzheimer’s disease (3xTg-AD). Formalin-fixed free-floating sections were subjected to immunostaining using antibodies against Iba-1 and β-amyloid (clone 6E10). Microglia were identified by brown signal and amyloid plaques by red signal. (**A**) Microglia in the healthy mouse brain at the level of hippocampus; (**B**) High magnification image of ramified microglia with a small cell body and fine extensive processes; (**C**) Activated microglia are present throughout the hippocampal section; (**D**) High magnification image of activated microglia surrounding amyloid plaque; (**E**–**H**) Morphological transformation of homeostatic microglia to disease-associated phenotypes, where their branches appear short, stubby and cells appear amoeboid.

Microglia are the principal neuroimmune cells of the CNS and constitute ~5–20% of total brain cells. They display regional differences in density and form an intricate network in the three-dimensional space of the brain parenchyma where they are in contact with neuronal and other non-neuronal cells, including astroglia, oligodendroglia, endothelial cells and mast cells [46]. Microglia are vigilant housekeepers and carry out three main homeostatic functions, namely sensing, housekeeping, and neuroprotection to safeguard CNS homeostasis [8]. In the healthy brain microglia are in a “resting” state and appear morphologically ramified with small cell bodies and fine, long branched processes (Figure 2A,B) [47]. Although referred to as ‘resting’, ramified microglia continuously scan the brain microenvironment every few hours for extracellular threats by extending and retracting their processes [24,48]. They are involved in immune surveillance and are responsible for the detection and elimination of threatening agents and ensure neuronal survival. When carrying out their homeostatic duties, microglia are in constant motion and display diverse phenotypes, however the significance of these changes had been difficult to ascertain, until recently.

#### 3.2.1. Homeostatic Microglia: Nurturers of the Brain

With the advent of cutting-edge technologies, such as next generation RNA sequencing (RNA-seq), research into the morphofunctional states of microglia has provided a high-resolution view of CNS innate immune cells, and dramatically accelerated our understanding of the transcriptional profile of these cells. RNAseq profiles revealed that microglial cells possess a unique molecular sensome or ‘homeostatic signature’ that is defined by changes in hundreds of genes, including *SLC2A5*, *P2YR12*, *P2RY13*, *TMEM119*, *GPR34*, *CX3CR1*, and *TREM2* that microglia use to scan the CNS microenvironment [49,50,51]. Using CX3CR1 (chemokine (C-X3-C motif) receptor 1)-GFP (Green Fluorescent Protein) mice, transcranial two-photon in vivo imaging has revealed microglial dynamics in a living rodent brain and shown that microglia processes sense pathological insults and migrate to the sites of injury to phagocytose and clear cellular debris, in turn, shielding the brain from damage [48]. In this process, microglia deploy gene products involved in chemotaxis (CX3CR1), phagocytosis (Triggering Receptor Expressed on Myeloid cells 2 (TREM2) and scavenger receptors) and synaptic pruning and remodeling (C1q and CX3CR1) [49]. It is plausible that disruption of this microglial housekeeping function could result in neuronal dysfunction and disease.

#### 3.2.2. Homeostatic Microglia: Sentinels of the Brain

As cellular soldiers of the innate immune system, microglia respond to very subtle alterations in their microenvironment. They express receptors for the classical neurotransmitters (e.g., α-amino-3-hydroxy-5-methyl-4-isoxazolepropionic acid (AMPA), γ-aminobutyric acid (GABA), kainate) and ion channels (K^+^, H^+^, Na^+^, Ca2^+^, Cl^−^). The functional state of microglia dictates the expression level of each of these ion channels [52]. Although microglia have been considered in the context of pathology, they play a crucial role in maintaining normal physiological functions. For example, microglia respond to injury by secreting several growth factors (e.g., fibroblast growth factor (FGF-2), insulin-like growth factor-1 (IGF-1)) and neurotrophic factors (e.g., brain-derived neurotrophic growth factor (BDNF), glial cell derived neurotrophic growth factor (GDNF) [53], NGF, NT-3) that provide trophic support to neurons and promote neuronal repair [50]. It has been shown that microglia promote learning-dependent synapse formation mainly through the secretion of BDNF in mice [54]. Similar to other tissue macrophages, microglia are the professional phagocytes of the CNS and defend not only against invading microbes but also are involved in removing debris from damaged cells and sculpting synaptic circuits in the developing brain [55,56]. Furthermore, they have been shown to phagocytose apoptotic cells during neurogenesis through the receptor tyrosine kinases—AXL and MER, belonging to the TYRO3, AXL and MER (TAM) receptor family [57]. Thus, microglial homeostatic function contributes to the maintenance of the CNS homeostasis.

Microglial cells possess a potent glutathione (GSH) antioxidant system and contain the highest concentrations of GSH, which might protect them from oxidative injury [58]. In addition, microglia express a large number of cytokines and chemokines as well as cytokine and chemokine receptors which have normal physiological functions, including neuronal migration (Regulated on Activation, Normal T Cell Expressed and Secreted, (RANTES)/chemokine (C-C motif) ligand 5 (CCL5), Stromal cell-Derived Factor-1 (SDF-1α)/chemokine (C-X-C motif) ligand 12 (CXCL12), cell proliferation (SDF-1α/CXCL12), Growth-regulated oncogene (Gro-α)/chemokine (C-X-C motif) ligand 1 (CXCL1) and synaptic activity (Gro-α/CXCL1, IL-8/CXCL8, chemokine (C-X-C motif) receptor 1 (CXCR1), chemokine (C-X-C motif) receptor 2 (CXCR2), chemokine (C-X-C motif) receptor 4 (CXCR4) [59]. Cytokines produced and released by microglia include IL-1, IL-6, IL-10, TGF-β, and TNF-α, which have neuroprotective functions at lower concentrations. It has been demonstrated that treatment of microglia with TGF-β produces anti-inflammatory molecules and downregulate the production of pro-inflammatory mediators [60]. Treatment of primary neuronal cultures or organotypic slice cultures with TGF-β protects neurons against excitotoxicity [61]. Taken together, it is clear that microglia secrete a plethora of growth factors, cytokines, and chemokines that support neuronal fitness.

## 4. Mast Cell Activation in Neurodegeneration

Mast cells are known for their role in allergic inflammation and anaphylactic reactions [37,62] but increasing evidence indicates that aberrant brain mast cell behavior may cause or exacerbate neuroinflammation and neurodegeneration [14]. As described in Section 3.1, mast cells in the CNS are uniquely positioned to orchestrate both vascular and neural responses. In the CNS, they are situated around blood vessels (Figure 1B,C) and reside close to other innate immune cells, astroglia, microglia (Figure 1D), and oligodendroglia, as well as neurons. Mast cells are equipped with a variety of surface receptors, which include IgG receptors, complement receptors, cytokine and chemokine receptors, pattern-recognition receptors (TLRs, NLRs) and purinergic receptors to recognize, PAMPs, DAMPs, and NAMPs. A rapid immune response is initiated by deploying a preformed and preactivated repository of pro-inflammatory mediators to eliminate the threat and safeguard CNS homeostasis.

### 4.1. Mast Cells: Guardians of Homeostasis in the Brain

Although they are often described in the context of pathology and disease, mast cells are likely important regulators of homeostasis, since mast cell mediators can have both beneficial and harmful effects depending on the context in which they are deployed. This may also be the case in the brain. For example, activated mast cells rapidly release a series of immunomodulatory molecules, such as histamine and TNF-α. In organotypic slice cultures and primary rat astrocyte-neuron co-cultures, exogenously added histamine was shown to protect hippocampal neurons against glutamate-induced excitotoxicity [63]. Neuroprotection was mediated by increased expression of astrocytic glutamate transporter-1 (GLT-1), probably due to reduction in extracellular glutamate levels. Mast cell-derived proteases and proteoglycans also might provide neuroprotection [64]. In a mouse model of ischemic injury, TNF-α was shown to promote the survival of hippocampal and striatal neurons, probably acting via its receptor (tumor necrosis factor receptor 2 (TNFR2)) [65]. The protective or detrimental effects of TNF-α might depend on the concentration and duration of release as well as receptor binding (TNFR1 vs TNFR2) [66]. Intracerebral mast cell secrete proteases, vasoactive molecules such as nitric oxide, lipid mediators, histamine, gonadotropin-releasing hormone and TNF-α which can increase BBB permeability by breaking down the tight junctions between brain endothelial cells [67]. Thus in pathological situations, mast cells appear to operate in a feed-forward mechanism; loss of BBB integrity might activate meningeal mast cells to recruit inflammatory cells to the CNS, leading to a vicious cycle of neuroinflammation [68]. These findings suggest that the beneficial or detrimental effects of mast cells to brain health might be dependent on several factors, including levels and time of cytokine release and the magnitude of the initial insult.

### 4.2. Mast Cells: Warriors of the Brain

Neuroinflammation is an important component of age-related neurodegenerative diseases and mast cells have been implicated in disease initiation and progression. Histopathology of post-mortem AD brain showed an increased number of tryptase-positive mast cells congregated around β-amyloid plaques compared with age-matched controls [69]. These findings were corroborated in the transgenic mouse model of AD, where an increased number of mast cells were found in the hippocampus and cortex prior to β-amyloid deposition, suggesting that mast cells are among the first immune cells to sense misfolded aggregated proteins and home to the site of pathology [70]. Furthermore, in vitro treatment of brain mast cells with β-amyloid peptide 25–35 activated Panx1 and Cx43 hemichannels, probably by increased Ca^2+^ influx and release of histamine [70]. Indeed, masitinib mesilate (AB1010), a selective tyrosine kinase inhibitor that targets c-kit was shown to reduce cognitive decline in AD patients when administered orally as an adjunct therapy to the standard-of-care treatment [71]. It is highly likely that the therapeutic effects are due to modulation of mast cell function and preservation of the BBB integrity. Currently, masitinib is under investigation in phase 2/3 clinical trials for the treatment of mild-to-moderate AD [72].

Although impaired BBB function and loss of BBB integrity is documented in laboratory animal models of PD and human PD patients, there is limited evidence for the direct involvement of mast cells in PD pathogenesis. Recent work by Kempuraj and colleagues has shed some light on the role of mast cells in PD. In vitro treatment of mouse bone marrow-derived mast cells (BMMCs) with 1-methyl-4-phenylpyridinium (MPP^+^), an active metabolite of the parkinsonian neurotoxin 1-methyl-4-phenyl-1,2,3,6-tetrahydropyridine (MPTP), activates BMMCs and enhances the release of chemokine MCP-1 (also known as chemokine (C-C motif) ligand 2 (CCL2)) and matrix metalloproteinase-3 (MMP-3) [73]. CCL2 is a well-known mast cell activator that activates murine mast cells to degranulate [37] and exposure of BMMCs treated with MPP^+^ to glia maturation factor (GMF), a pro-inflammatory protein, further enhances CCL2 release [74]. In vivo evidence for mast cell involvement in PD was recently obtained from the MPTP mouse model of dopamine neuron degeneration [75]. Mast cells recruited into the substantia nigra expressed transglutaminase 2 (TG2) and mast cell markers (c-kit, tryptase and FcεR1), as well as secreted increased levels of mast cell mediators (TNF-α, histamine and leukotrienes), accompanied with loss of dopamine neurons. These effects were absent in TG2-deficient mice, but recapitulated in TG2-deficient mice that had received adoptively transferred wild-type BMMCs. Interestingly, increased bioactivity of TG2 and mast cell markers was also seen in the serum of PD patients as compared to healthy subjects, suggesting that TG2 may be a useful biomarker [75]. The exact molecular mechanisms by which mast cells contribute to neuroinflammation and neurodegeneration are not completely understood but likely includes a complex set of communication signals between mast cells and other brain cells such as microglia.

## 5. Microglial Activation in Neurodegeneration

Activated microglia with different morphologies congregate around the sites of neurodegeneration (Figure 2C,D) and recent research has focused on the molecular characterization of these microglia in disease progression. Because microglia are equipped with a unique repertoire of sensors of pathology on their plasma membrane (e.g., TLRs), any deviation from homeostasis leads to their activation and transition into different phenotypes [11,76]. A growing number of recent studies have shown that under pathological conditions, microglia activates a defense program and transition from the ‘homeostatic state’ into the ‘disease-associated’ state and during disease progression might “lose control” and transition into a ‘neurodegenerative-disease state’ and become dysfunctional and destructive to the CNS [50,77]. Microglia from each disease-associated state express a unique transcriptional signature [78,79]. Whether microglia exacerbate or inhibit disease progression is still being actively debated, with both beneficial and detrimental roles ascribed to these cells in neurodegeneration [80].

### 5.1. Disease-Associated Microglia: Sentinels of the Brain

As an important first line of defense, microglia readily respond to injury or immunologic stimuli [24], become activated and transforms morphologically and biochemically into a hypertrophic or “amoeboid” immune phenotype (Figure 2E–H). In addition, activated microglia with homeostatic mRNA signatures migrate to the site of pathology and produce high levels of pro-inflammatory mediators such as cytokines (IL-1, IL-2, IL-6, TNF-α, arachidonic acid metabolites, and RNOS through activation of inducible nitric oxide synthase (iNOS) and nicotinamide adenosine dinucleotide phosphate (NADPH) oxidase system [17,81]. Low to moderate concentrations of RNOS are known to activate the antioxidant defense systems, such as NF-E2-related factor 2 (Nrf2), which in turn, upregulates the expression of catalase, superoxide dismutase (SOD), glutathione peroxidases (GPX), peroxiredoxins (PRX) and hemeoxygenases (HO) [82]. These inflammatory responses are pro-resolving and beneficial for the host as microglia eliminates the injurious agent, initiates repair and restores tissue homeostasis; however inflammation can drive disease progression when it fails to subside [83].

### 5.2. Disease-Associated Microglia: Warriors of the Brain

Under pathological state, microglia continuously release excessive amounts of pathogenic pro-inflammatory mediators, excitatory amino acids (e.g., glutamate), complement activation products, proteolytic enzymes and RNOS, which, in turn, generates oxidative stress [84]. Exposure of in vitro neuronal-glia cultures to glutamate resulted in oxidative stress and neurotoxicity [85]. Microglia respond to misfolded β-amyloid by producing RNOS, notably superoxide anions via the NADPH oxidase. These reactive species can be inactivated by extracellular SOD to produce hydrogen peroxide, which can be further detoxified by the antioxidant defense systems, namely SOD, GPX, PRX, and HO [82]. Superoxide can also react rapidly with nitric oxide to produce peroxynitrite, a potent oxidizing and nitrating agent [86]. Interestingly, NADPH oxidase activity is increased in activated microglia in early stage AD patients as compared to age-matched controls [87].

Oxidative stress has long been suspected to contribute to the lesions in acute and chronic neurodegenerative diseases [82]. Evidence of oxidative stress damage in patients with neurodegenerative diseases include the presence of proteins that have been modified by nitration or glycation, and the existence of low molecular weight compounds that have been oxidized and nitrated such as 4-hydroxynonenal, 3-nitrotyrosine, 8-hydroxy-deoxyguanosine, and peroxidated lipids [82]. Inflammation in AD is characterized by the accumulation of activated microglia at the sites of β-amyloid deposition [17,88]. There is ample in vitro evidence that upon activation with β-amyloid, microglia produce pro-inflammatory mediators such as cytokines (IL-1, IL-6, and TNF-α), chemokines (IL-8, MIP-1, and MCP-1), cyclooxygenase-2 [89] and RNOS that are neurotoxic [17,90]. The degeneration of dopamine neurons in PD is also associated with a massive accumulation of microglia [91,92]. The accumulation of RNOS, pro-inflammatory lipids and cytokines in the serum, cerebrospinal fluid (CSF) and post-mortem brains of patients with neurodegenerative diseases further support a role of uncontrolled prolonged neuroinflammation [93].

### 5.3. Disease-Associated Microglia: Enemies within the Brain

Hallmarks of neurodegenerative diseases include the presence of microglia around the sites of β-amyloid (Figure 2C,D) and α-synuclein deposits [17,94]. These findings were recapitulated in murine models of AD where microglia were shown to rapidly migrate and accumulate around β-amyloid plaques. However, instead of internalizing and removing β-amyloid from plaques, microglia appear to contribute to their morphological and chemical evolution facilitating the conversion of existing soluble and oligomeric β-amyloid peptides within plaques to the fibrillar form [95]. Similarly, microglia surround extracellular α-synuclein deposits in the brains of animal models of PD and post-mortem PD patients [96,97].

As neurodegeneration progresses, microglia progressively switch their transcriptional program to transition from a basal homeostatic state to disease-associated phenotypes. In a recent study, Keren-Shaul et al., used single-cell RNA-seq and identified distinct populations of microglia associated with β-amyloid plaques in transgenic 5XFAD mice based on their unique transcriptional signatures [78]. Microglial populations showed specific changes in gene expression profiles, including reduced expression of homeostatic genes (*CX3CR1*, *P2RY12*, *TREM2*) and increased expression of phagocytosis genes (triggering receptor expressed on myeloid cells 2 (TREM2), AXL, lysosomal genes (*CTSB* and *CTSD*) and lipid metabolism genes (*APOE*, *LPL*)—referred to as ‘disease-associated’ state. It is important to determine if disease-associated microglial populations are neuroprotective or deleterious to better understand their role in disease pathology [98]. Both in rodents and humans, the TREM2-APOE pathway was identified as a major switch that mediated transition from a homeostatic to a neurodegenerative disease-associated phenotype [79]. More recently, Mathys et. al., used single-nucleus RNA-seq to study the cellular heterogeneity in brain cells of AD patients [99]. The transcriptome of brain cells revealed clear differences between neurons and glial cells during AD progression. While a repression of homeostatic signature was seen in neurons, microglia showed an activated phenotype with increased expression of major histocompatibility complex-II (MHC-II) protein human leukocyte antigen-DR, apolipoprotein E (ApoE), TREM2 and CD14, a co-receptor for TLR4. Furthermore, this study has also revealed distinct molecular signatures between male and female AD patients.

## 6. Mast Cell-Microglia Interactions in Neurodegeneration

Mast cells and microglia are found in close proximity to each other in the CNS, facilitating active communication. Mast cells likely use their surface receptors, adhesion molecules and ‘mast cell mediators’ to engage in a complex cross-talk with other brain-resident cells, which could be both unidirectional and bidirectional. Increasing evidence suggests that the initiation and propagation of neuroinflammation relies on the interactions between these cell types. Mast cells also express several costimulatory and inhibitory surface molecules that allows them to communicate with other immunocompetent cells, such as T-cells and B-cells, positioning them as a main bridge between innate and adaptive immunity [37,100].

Activated brain mast cells release histamine that can cause phenotypic changes and activation of microglial cells [101]. Exogenously added histamine triggered activation of cultured primary cortical microglia, murine N9 microglia and hippocampal organotypic slice cultures to secrete TNF-α and IL-6 and RNOS [102,103]. Although histamine stimulated microglial cell motility in control microglia, histamine inhibited microglial migration and IL-1β release in LPS-stimulated microglia, suggesting a dual role of histamine in modulating microglia-induced inflammatory responses [102,104]. Histamine exerts its functions by signaling through four types of G-protein coupled receptors, namely Histamine 1 receptor (H1R), H2R, H3R, and H4R, which are expressed on innate immune cells, neurons and endothelial cells [105]. In these cultures, the anti-inflammatory effects of histamine were mediated by activation of H4R which involved α5β1 integrin, p38 and protein kinase B (AKT) signaling to restrain exacerbated microglial responses in neuroinflammation [102]. All four types of histamine receptors are expressed by microglial cells and can modulate microglia-mediated neuroinflammation [104]. Rocha SM et. al., directly injected histamine into the substantia nigra of mice and studied its effects on microglial activity and dopaminergic neuron survival [106]. In accordance with other studies, histamine induced microglial activation and dopaminergic neuronal toxicity via H1R activation, probably through NADPH oxidase dependent oxidative stress signaling pathways and microglia phagocytosis. Altogether, these studies show that histamine per se triggers a pro-inflammatory response and under inflammatory conditions, histamine activates an anti-inflammatory response, dampens microglial-induced inflammation and is associated with neuroprotection.

Similar to peripheral mast cells, brain mast cells are also known to secrete proteases, such as tryptase. Exposure of primary microglia to mast cell-derived tryptase stimulated microglia to subsequently secrete TNF-α and IL-6 and RNOS. These effects were mediated by protease-activated receptor-2 (PAR-2) signaling via activation of mitogen-activated protein (MAP) kinase (Erk and p38) and NF-kappa B (NF-kB) pathways [68]. Furthermore, PAR-2 activation induces the expression of ATP-sensitive ionotropic P2X4 receptors on microglia and exposure to ATP leads to secretion of BDNF, a potent trophic factor [107]. The presence of functional P2X4 receptors are also expressed by human mast cell lines [108]. Since mast cells play a pivotal role in neuroinflammation, it is important to determine the exact molecular mechanisms employed by activated mast cells and microglia and their role in disease progression.

Mast cell-derived pro-inflammatory cytokines such as CCL2, TNF-α and IL1β can also influence microglia activation. To recapitulate in vitro mast cell-glia-neuron crosstalk during neuroinflammation, mast cells were cocultured with mixed cultures of neuron and glia or enriched cultures of neurons or astroglia and challenged with MPP^+^ or GMF or mast cell proteases [109]. Mast cells cocultured with glia had increased production of CCL2 and IL-33, highlighting the importance of mast cell-glia coupling and their role in neuroinflammation [110]. Increased CCL2 levels have been demonstrated in AD patients which is associated with accelerated cognitive decline and AD progression [111]. CCL2 expression altered β-amyloid phagocytosis, supporting the notion that microglial phagocytosis could be regulated by mast cells [112]. Although there is no direct evidence of CCL2 expression in the brain of PD patients, increased serum levels of CCL2 have been reported [113]. Recently, two polymorphisms have been reported in the promoter region of CCL2 which are associated with an increased risk of PD [114]. The exact role of CCL2-CCR2 axis in regulating mast cells and microglia in neurodegenerative diseases is still not well understood.

Mast cell degranulation has been shown to activate microglia. Stereotaxic injection of a mast cell degranulation compound 48/80 (C48/80) and activator of the mas-related G protein-coupled receptor Mrgpr [115] in the hypothalamus of rats induced mast cell degranulation, production of pro-inflammatory cytokines and microglia activation [103]. These effects were mediated by activation of MAP kinase and AKT pathways and an increased protein expression of H1R, H4R, PAR-2 and TLR4 on microglial cells. Treatment with a mast cell stabilizer disodium cromoglycate (cromolyn), inhibited microglial activation and downstream signaling, suggesting mast cell involvement. Most importantly, C48/80 had no effect on microglial activation in mast cell-deficient Kit^w-sh/w-sh^ mice. These data support the notion that stabilization of brain mast cells during neuroinflammation could be a new therapeutic strategy to restrain microglial hyperactivity. Tranilast has been used to inactivate the NLR family pyrin domain containing 3 (NLRP3) inflammasome, yet is also used as an anti-allergy medication as a “mast cell stabilizer” [116] suggesting that its effect in the brain may also modulate mast cell functions via the inflammasome.

### NLRP3 Inflammasome: Common Sensor in Microglia and Mast Cells

**Figure 3:**
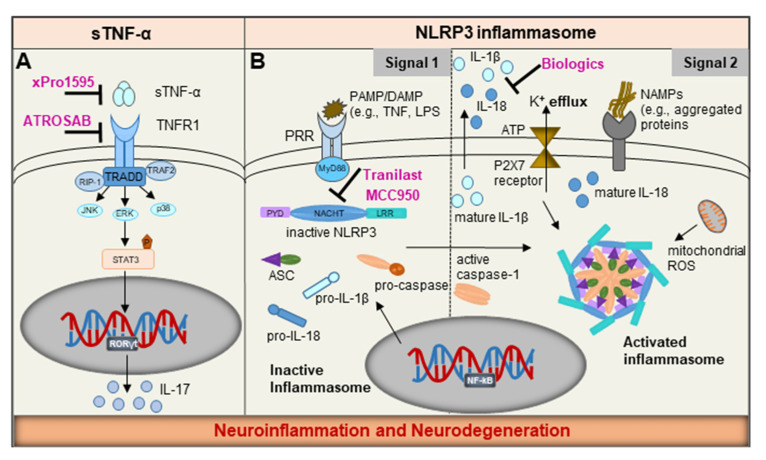
Schematic representation of potential therapeutic approaches targeting common pathways in mast cells and microglia. Tumor necrosis factor (TNF) signaling and the NLRP3 inflammasome, represent the two common pathways that can be targeted to dampen inflammation. (**A**) ATROSAB, a TNFR1-specific antagonist and xPro1595, a neutralizing antibody specifically inhibit soluble TNF-α; (**B**) Biologics, namely recombinant IL-1β receptor antagonist (Anakinra), IL-1β neutralizing antibody (Canakinumab) and a soluble decoy receptor that binds IL-1β (Rilonacept) are in clinical use. Tranilast (Rizaben) and MCC950 have been identified as specific inhibitors that binds to the NOD domain (also referred as the NACHT domain) of the NLRP3 and inhibit inflammasome assembly and activation, in turn, inhibiting the vicious cycle of neuroinflammation and neurodegeneration.

In the sections above, the individual signals that activate either mast cells or microglia were described. However, it is possible that mast cells and microglia can be activated by the same signal and function concurrently to orchestrate inflammation in the brain. Both mast cells and microglia contain key signaling hubs in the cytoplasm, called inflammasomes [117,118]. The main function of inflammasomes is to detect and eliminate a variety of stimuli including PAMPs, DAMPs, or NAMPs by subsequent proteolytic cleavage of pro-IL-1β and pro-IL-18 to generate their bioactive forms and secretion of IL-1β and IL-18, respectively (Figure 3B). The NLRP3 is the best-studied inflammasome which consists of three major components: (i) the NLR protein as sensor, (ii) the Apoptosis-associated Speck-like protein containing a Caspase-activating and recruitment domain (ASC) as adaptor, and (iii) the protease caspase-1 as effector. The adaptor, ASC acts as a direct bridge between the sensor and the downstream effector caspase-1 and plays a central role in the assembly of the NLRP3 inflammasome machinery [119,120].

The activation and assembly of NLRP3 inflammasome is under tight regulation and occurs by a two-signal process, consisting of a priming signal and an activation signal (Figure 3B) [121]. The priming signal is delivered by stimulation of TLRs on host cells and is associated with NFĸB-dependent transcription of pro-IL-18, pro-IL-1β and NLRP3. The second signal is elicited by multiple stimuli, including NAMPs released from damaged or degenerating neurons, abnormally aggregated β-amyloid and α-synuclein, ionic flux (e.g., efflux of K^+^ or Cl^−^ ions or influx of Ca^2+^ ions), high glucose, exogenous ATP, reactive oxygen species from mitochondrial damage or lysosomal proteases [17]. Upon activation, NLRP3 inflammasome assembles by recruiting ASC which rapidly forms dimers and polymerizes to assemble into large filamentous structures known as ASC-specks, a macromolecular form of the inflammasome which activates caspase-1 [122]. The ASC-specks are released into the extracellular space, where they act as signaling platforms to activate free precursor IL-1β or are phagocytosed by myeloid cells leading to further activation of caspase-1 and release of IL-1β or IL-18 [123]. The overactivated NLRP3 inflammasome can constitutively release cytokines resulting in pyroptosis, an inflammatory form of cell death, which can exacerbate chronic inflammatory responses.

Increasing number of preclinical and human clinical studies have shown that the NLRP3 inflammasome can be activated by several NAMPs in chronic neurodegenerative diseases, including AD and PD [22,124]. The binding of β-amyloid to TLR2 on the microglia triggers NLRP3 activation, thereby releasing IL-1β. Accordingly, components of NLRP3 inflammasome such as increased levels of cleaved caspase-1 and IL-1β were detected in the serum of early AD patients or patients with mild cognitive impairment (MCI) as compared to non-demented and age-matched controls [125]. More importantly, ASC bound β-amyloid was also found in the post-mortem AD brain tissue [123]. These findings suggest that NLRP3 activation represents an early biomarker of AD. A large number of studies conducted in the APP/PS1 transgenic mice, which recapitulates most features of AD pathology, has consistently concluded that β-amyloid deposition, microglial NLRP3 activation and IL-1β release is an early feature of AD progression [124]. The evidence for ASC-speck release by microglial pyroptosis and its spread by a ‘prion-like’ mechanism was obtained in the APP/PS1 mouse brains. ASC-specks released by microglia were able to cross-seed β-amyloid in the surrounding microglia, in turn, leading to propagation of neuroinflammatory responses in AD progression [122,123,126]. Interestingly, APP/PS1 mice that lacked components of the NLRP3 apparatus showed a reduction in β-amyloid burden, possibly due to increased microglial phagocytosis and reduction in cognitive decline [127]. Moreover, treatment with MCC950, a specific inhibitor of the NLRP3 that inhibits NLRP3-induced ASC oligomerization, promoted non-phlogistic clearance of β-amyloid and ameliorated cognitive impairment [128]. These studies revealed NLRP3 as a potential therapeutic target for treating early-stage AD.

More recently, the NLRP3 inflammasome has also been shown to play an important role in PD pathogenesis. Hallmarks of inflammasome activation, i.e., cleaved caspase-1, IL-1β NLRP3 and ASC-specks were found in the postmortem PD brain tissues and in plasma of PD patients compared to age-matched controls, suggesting systemic increase in inflammasome activation [129,130,131]. Furthermore, systemic increases in NLRP3 activation shows a positive correlation with motor function decline and PD progression [132]. A strong association between α-synuclein levels and microglial NLRP3 inflammasome activation accompanied with dopaminergic neuron toxicity has been shown in several animal models of PD, including the MPTP mouse model of PD [129]. Oral administration of MCC950 reduced pro-inflammatory cytokines and attenuated dopaminergic neuron loss [129]. Furthermore, NLRP3-deficiency abolished MPTP-induced microglial activation, caspase-1 and subsequent IL-1β release, suggesting that NLRP3 pathway plays a pivotal role in MPTP-induced neurodegeneration [133]. These results indicate that therapeutic targeting of the NLRP3 pathway has the potential to slow down or halt PD progression.

Although, to date, no reports have investigated the expression of the NLRP3 inflammasome in brain mast cells, peripheral tissue mast cells from cryopyrin-associated periodic syndrome (CAPS) patients were, for the first time, shown to express functional inflammasomes and secrete IL-1β [134]. Notably, mast cells from the skin of CAPS patients expressing the disease-associated NLRP3 mutations constitutively produced IL-1β and mediated histamine-independent urticarial rash. Furthermore, these patients responded to IL-1 receptor antagonist therapy rather than antihistamines, suggesting that urticaria in CAPS patients is mediated by IL-1β [134]. Seminal work from Melissa Brown’s research team has also shown that mast cell inflammasome is a critical mediator of inflammation in the meninges and regulates disease severity in a rodent model of multiple sclerosis [135]. The meningeal mast cells, which lie outside the BBB, release IL-1β and licenses T-cell pathogenicity [135]. These studies support the notion that mast cells are principal drivers of inflammatory responses. Although the direct role of NLRP3 inflammasomes in brain mast cell activation and function in AD and PD is lacking, it is not unreasonable to postulate that brain mast cells may play an important role in neuroinflammation and neurodegeneration.

## 7. Emerging Therapeutics

Currently, there is no effective disease-modifying therapy available for patients with neurodegenerative diseases. Therefore, the development of interventions aimed at slowing disease progression represents a critical unmet need. Based on accumulating evidence from recent investigations, molecular targets that can be precisely targeted in both brain mast cells and microglia to inhibit inflammatory signaling are discussed.

### 7.1. NLRP3 Inflammasome—A ‘Druggable’ Target

The NLRP3-inflammasome is constitutively activated in response to a variety of sterile inflammatory triggers making it a highly relevant druggable target (Figure 3B). The specific targeting of the NLRP3 and ASC represents an innovative strategy to extinguish the fire of inflammation. Thus far, only the inflammasome effector molecule IL-1β has been targeted by protein-based therapies such as recombinant IL-1β receptor antagonist (Canakinumab), IL-1β neutralizing antibody (Anakinra) and a soluble decoy receptor that binds IL-1β (Rilonacept) [136]. While these therapies block IL-1β, the observed effects are likely peripheral as there is no evidence of BBB penetration. Moreover, IL-1β is produced by inflammasome-independent pathways and IL-1β blockade may lead to unintended immunosuppression and an increase in infections [137]. To date, MCC950 has been identified to be the most selective and potent small-molecule inhibitor of NLRP3. It interacts with the Walker B motif within the central NOD domain (also referred as the NACHT domain) of NLRP3, and curtails its activation by blocking ATP hydrolysis and blunting the assembly and activation of the NLRP3 inflammasome [138]. More recently, Tranilast (Rizaben), an anti-allergy drug derived from tryptophan, was identified as a specific inhibitor of NLRP3 inflammasome. Tranilast was shown to directly bind NLRP3 within its NACHT domain thereby inhibiting NLRP3-NLRP3 interaction and subsequent oligomerization [139]. Because of its potent anti-inflammatory properties, Tranilast has been clinically used in patients and shown to inhibit IgE-induced histamine secretion from mast cells [116]. Although NLRP3 activation has not been demonstrated in brain mast cells, it is possible Tranilast crosses the BBB [140] and inhibits NLRP3 in brain mast cells. Thus, precision targeting of NLRP3 components might prove to be safer and more effective strategy for treating sterile inflammatory diseases, including neurodegeneration.

### 7.2. Precision Targeting of Soluble TNF-α

As discussed above, TNF-α is a pleiotropic cytokine secreted both by microglia and mast cells during an inflammatory response. A variety of TNF-α antagonists, including adalimumab (Humira), etanercept, infliximab (Remicade), and certolizumab have been successfully used for treating several inflammatory diseases [141]. However, complete inhibition of TNF-α is associated with severe side-effects since it adversely impacts the ability of the host to protect itself against infections [142]. TNF-α is expressed as a transmembrane protein (tmTNF-α) that is cleaved by ADAM17 to release soluble TNF-α (sTNF-α); sTNF-α is known to bind TNFR1 and tmTNF-α binds to TNFR2. It is well established that TNFR1 predominantly mediates pro-inflammatory signaling, whereas TNFR2 mediates neuroprotective effects and promotes tissue homeostasis [66]. Hence, specific inhibition of sTNF-α seems to be a rationale therapeutic strategy to dampen the aggravated neuroinflammatory responses in neurodegenerative diseases [143]. Accordingly, dominant-negative TNF variants were engineered as selective inhibitors that blocked binding of sTNF-α to its receptor and the TNFR1-mediated signaling [144].

Selective TNF variants have been shown to have neuroprotective effects in several animal models of neuroinflammation and neurodegeneration. Intranigral delivery of XENP345, a PEGylated formulation of recombinant dominant-negative TNF, directly into the striatum attenuated dopaminergic neuron loss and amphetamine-induced rotational behavior as well as preserved striatal dopamine levels in the 6-hydroxydopamine (6OHDA)-induced rat model of PD [143]. Moreover, systemic delivery of XPro^®^-1595 also significantly reduced markers of neuroinflammation and dopamine neuron loss, suggesting that XPro-1595 might have disease-modifying potential in PD progression [145]. Plasma levels of 1–8 µg/mL and CSF levels of 1–6 ng/mL were reported and the therapeutics effects were likely mediated by the ability of XPro^®^-1595 to cross the BBB and engage its target in the CNS. Similar effects following systemic delivery of XPro^®^-1595 were also observed in the 5xFAD transgenic mouse model of AD. Selective targeting of sTNF-α mitigated AD pathology i.e., reduced hippocampal β-amyloid load, dampened neuroinflammatory responses and delayed neuronal dysfunction [146]. Importantly, neutralization of sTNF-α prevented synaptic deficits in the TgCRND8 transgenic model of early AD [147]. ATROSAB, a humanized antagonistic anti-TNFR1 antibody also blocked TNFR1-mediated signaling [148]. These studies support the clinical development of neutralizing antibodies to sTNF-α and antagonistic antibodies to TNFR1 as potential immunomodulators for the treatment of neurodegenerative diseases (Figure 3A).

## 8. Concluding Remarks and Perspectives

**Figure 4:**
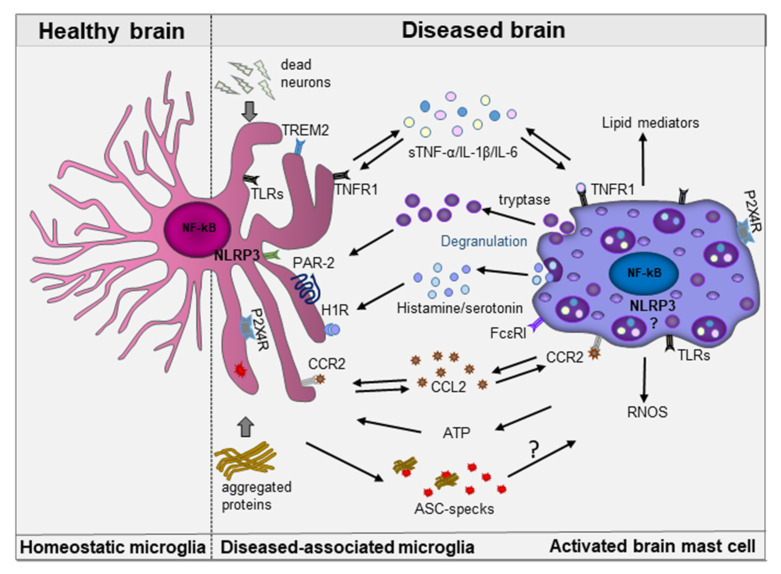
Schematic illustrating mast cell-microglia communication in the diseased brain. Activated mast cells and microglia can interact in a unidirectional or bidirectional manner using a range of preformed and newly synthesized pro-inflammatory molecular mediators, such as cytokines (TNF-α, IL-1β, IL-6) chemokines (CCL2), lipids, histamine, serotonin, tryptase, adenosine, and RNOS. These mediators can act in an autocrine or paracrine manner. Each cell type is armed with an array of sophisticated receptors, for example TLRs, TREM2, H1R, PAR-2 TNFR1, FcεRI, CCR2, and P2X4R that decode the molecular information resulting in functional changes, thereby leading to resolution or amplification of the inflammatory responses. Although activation of the NLRP3 inflammasome is well-documented in microglia, its activation in brain mast cells is not yet established. ASC-specks released by microglia can bind β-amyloid and further amplify inflammatory responses leading to a vicious cycle of neuroinflammation and neurodegeneration, however its effect on mast cells is unknown.

Age-related neurodegenerative diseases are increasingly recognized as a major public health challenge in the 21st century and novel treatment strategies are urgently needed. In the past few decades, much attention was focused on understanding the selective loss of specific neuronal populations and their vulnerability to identify neuroprotective strategies. However, innate immune cells, including mast cells have recently received recognition and emerged as potential contributors to neuropathology, mainly by losing their homeostatic functions and gaining pathogenic functions. Although the exact molecular mechanisms involved in mast cell-microglia communication are only beginning to be decoded, it is becoming evident that this cross-talk might contribute to neuroinflammation and neurodegeneration. Based on our current understanding of the literature a model depicting some of the complex interactions occurring between disease-associated microglia and activated mast cells under chronic neurodegenerative diseases is presented in Figure 4.

It still remains unresolved whether chronic activation of the innate immune system plays a beneficial or detrimental role in CNS repair or regeneration. Notably, much of the work documenting a neurotoxic or neuroprotective role of microglia and mast cells has been carried out in human transformed cell lines and rodent models of disease. There are striking differences between rodent and human microglia that need to be considered. For instance, aged human microglia express higher levels of genes signatures related to immune function and neuroinflammatory pathways compared to their rodent counterparts [149]. The interspecies age-related divergence between humans and rodent microglia and limitations in faithfully recapitulating the in vivo neuroinflammatory processes supports the need for the development and use of new translational models. The human induced pluripotent stem cell (iPSC)-based technologies have emerged as a powerful toolkit that beholds great promises for CNS disease modeling [150]. Future studies using human iPSC-derived mast cells and microglia grown as 3-dimensional co-cultures or organoids with other neural cells are justified to decode the dialogue between these cell types in regulating homeostasis and dysfunction that contributes to neurodegeneration.

The complexity of the CNS microenvironment has been a barrier to our understanding of neuropathological mechanisms. Recent advances in RNA-seq methods has generated high-throughput gene expression data and microglial molecular signatures at a single-cell resolution and improved our understanding of microglial disease phenotypes. Although this data is powerful, it is not spatially or temporally defined, and we know very little about how cells migrate and communicate with one another in different regions of the brain in real-time. Spatial transcriptomics, which obtains transcriptional signatures in tissue sections, may help further decipher the interactions between various cell types of the CNS innate immune system. Although mast cells clearly participate in the initiation and perpetuation of neuroinflammation, our understanding of the brain mast cells in the context of health and disease is still obscure. Studies on brain mast cells have been limited due to the paucity of these cells in the brain (as compared to microglia and astroglia) and the difficulty in isolating intact mast cells from brain tissue. It would be desirable to use spatial transcriptomics to obtain molecular data for brain mast cells which could provide valuable insights into the role of mast cells during disease progression.

While still in their infancy, the advent of new technologies have improved our understanding of the role of the innate immune cells in the CNS. This knowledge will aid in the design of better treatment strategies that target mast cells and microglia in the brain to combat the epidemic of neurodegenerative diseases.

## Data Availability

Not applicable.
